# Correspondence to “Prediction of severe retinopathy of prematurity in 24–30 weeks gestation infants using birth characteristics”

**DOI:** 10.1038/s41372-021-01150-2

**Published:** 2021-07-24

**Authors:** Aldina Pivodic, Ann Hellström

**Affiliations:** Department of Clinical Neuroscience, Institute of Neuroscience and Physiology, Sahlgrenska Academy, University of Gothenburg, Gothenburg, Sweden

**Keywords:** Risk factors, Prognosis

## To the Editor:

We read with great interest Journal Club publication entitled *Prediction of severe retinopathy of prematurity in 24–30 weeks gestation infants using birth characteristics* by Dr. R. E. Zackula and Dr. T. S. Raghuveer [1]. We are grateful for the thorough review of DIGIROP-Birth, our prediction model for ROP treatment (ROPT), and for having its appropriateness evaluated by the newly developed PROBAST instrument assessing potential risks of bias [2–4]. Below we provide justifications to the raised questions with highest concern.

## Were all inclusions and exclusions of participants appropriate? (development and validation)

DIGIROP-Birth was based on 6947 infants born 2007–2017 at gestational age (GA) 24–30 weeks included in SWEDROP, the Swedish ROP registry. Of those, 289 (4.2%) had ROPT. From the development group, 94/7041 (1.3%) infants were excluded for missing data or date inconsistencies, 3 had ROPT. GA at birth and sex were similarly distributed in the excluded vs development group, 28.4 (SD 1.8) vs 28.3 (SD 1.9) weeks, and 47.9 vs 45.1% girls.

SWEDROP does not include race/ethnicity. A thorough validation of a model is a prerequisite for clinical implementation. If required, the model selection and/or parameter estimates might be re-evaluated for a specific population or clinical setting.

## Were there a reasonable number of participants with the outcome? (validation)

Although separately evaluated in our publication, 153/2122 (7.2%) infants had ROPT in the Swedish, German and US validation datasets. Per the PROBAST instrument, validation with ≥100 events is recommended [4].

## Were participants with missing data handled appropriately? (development and validation)

Reducing the effective sample by 1% (3/292 excluded events) and having no indication of infant selection in the excluded group, biased estimates were not expected. Internal validation including cross-validation and calibration plots, and external validations were affirmative.

## Was selection of predictors based on univariable analysis avoided? (development)

DIGIROP-Birth aimed to include few well-known risk factors available for all infants at birth; GA, sex and birth weight (BW) (*z*-score). Hence, univariable analyses were not required. The model was consecutively extended, starting with GA.

Concern was raised regarding multicollinearity for GA and BW *z*-score. We expect high correlation between GA and BW, *r* = 0.79 in this cohort. However, BW *z*-score extracts rest of the immaturity effect beside GA (and sex), *r* = −0.06. Therefore, we did not expect multicollinearity problem.

## Were relevant model performance measures evaluated appropriately? (validation)

In the DIGIROP-Birth publication the optimal cut-offs were not investigated. We identified cut-offs in our publication for the extended model incorporating ROP progression data (DIGIROP-Screen), proposing a clinical decision support tool [5]. Pre-specified cut-offs were defined using development group and 100% sensitivity. Applying those cut-offs on DIGIROP-Birth external validation cohort, sensitivity 149/153 (97.4%) and specificity 886/1969 (45.0%) were achieved. Further improvement of DIGIROP-Birth adding other known neo- and perinatal risk factors is planned in near future. Including more variables in a model increases the need for consideration of robust regression techniques that might reduce the potential risk for under- and overfitting.

We sincerely encourage and are thankful for all the efforts put on the validations and discussions of our work.
